# Single molecule imaging of protein aggregation in Dementia: Methods, insights and prospects

**DOI:** 10.1016/j.nbd.2021.105327

**Published:** 2021-06

**Authors:** John S.H. Danial, David Klenerman

**Affiliations:** a Yusuf Hamied Department of Chemistry, University of Cambridge, Cambridge, United Kingdom; b UK Dementia Research Institute, Department of Clinical Neurosciences, University of Cambridge, Cambridge, United Kingdom

**Keywords:** Single molecule microscopy, Protein aggregation

## Abstract

The aggregation of misfolded proteins is a fundamental pathology in neurodegeneration which remains poorly understood due to its exceptional complexity and lack of appropriate characterization tools that can probe the role of the low concentrations of heterogeneous protein aggregates formed during the progression of the disease. In this review, we explain the principles underlying the operation of single molecule microscopy, an imaging method that can resolve molecules one-by-one, its application to imaging and characterizing individual protein aggregates in human samples and *in vitro* as well as the important questions in neurobiology this has answered and can answer.

## 1 Bridging the knowledge gap in dementia with single molecule imaging

The aggregation of misfolded proteins plays a critical role in most neurodegenerative diseases (Goedert and Spillantini, 2006; Koffie et al., 2009; Goedert, 2015; Chiti and Dobson, 2006; Dobson, 2003; Benilova et al., 2012; Cleary et al., 2005; Haass and Selkoe, 2007; Knowles et al., 2014; Soto, 2003). Despite its central role in brain impairment, this complex pathway remains poorly understood at the molecular level (Birks and Harvey, 2006; Rolinski et al., 2012; Emre et al., 2010; Cummings et al., 2017; De Strooper, 2014). The state of the art in dementia research has largely focused on differences in the atomic structure of filamentous aggregates extracted from the *post mortem* brains of healthy and diseased individuals (Schweighauser et al., 2020; Falcon et al., 2019; Zhang et al., 2020), functional brain imaging at late stages of the disease (Schuff et al., 2009; Sabuncu et al., 2011), coarse characterization of the aggregates using ensemble techniques and *in vitro* studies which allow the aggregation proteome to be studied in isolation but without clear connection to disease pathology in humans (Flagmeier et al., 2017; Meisl et al., 2016; Shammas et al., 2015). Due to the combined lack of spatial resolution and statistical inference as well as the nanoscopic size of these aggregates and their heterogeneity and low abundance *in vivo*, none of the above methods bridge the gap between the molecular mechanisms and pathophysiological events characterizing the disease. Available drugs are designed to target single proteins, one-at-a-time, whilst neglecting the composition, structure and interaction partners of each aggregate which render the disease highly complex for treatment.

A quarter century of research in dementia and we still do not understand what are the main constituents of each aggregate, how does each aggregate interact with other protein machineries and which aggregates induce neuronal death and brain disease and by what mechanisms. Without answers to these fundamental questions, effective treatment is not foreseeable. The complex interactome formed by the aggregation proteome and its action over a remarkably broad range of spatiotemporal temporal scales requires the use of cutting edge nanoscale tools to visualize and characterize individual aggregates. Single molecule microscopy can weigh, count, size, track and resolve single molecules in action and on the nanoscale (Betzig et al., 2006; Ulbrich and Isacoff, 2007; Dahan et al., 2003; Szymborska et al., 2013; Dyla et al., 2017). Developments in the last decade have disclosed the complexity of the cellular milieu; now, we can see transporters translocating nutrients (Fitzgerald et al., 2019), endocytic machinery convening to regulate endocytosis (Mund et al., 2018) and apoptotic proteins initiating cell death (Salvador-Gallego et al., 2016). These intricate biological machines, and many more, can be imaged one at a time. In this review, we discuss single molecule methods, the principles underlying their operation, methodological challenges in their application to dementia research, the biological insights it had disclosed to date and its promise to unravel the molecular mechanisms underpinning neurodegeneration.

## 2 Single molecule microscopy of protein aggregates

In this section, we discuss how single molecule imaging can be used to resolve the nanoscopic soluble protein aggregates secreted *in vivo* as well as measure their surface hydrophobicity and composition. In addition, we describe a non-imaging based method with single molecule sensitivity which was extensively used in understanding the out-of-equilibrium, kinetic properties of recombinant aggregates.

### 2.1 Principle of single molecule localization microscopy

**Fig. 1:**
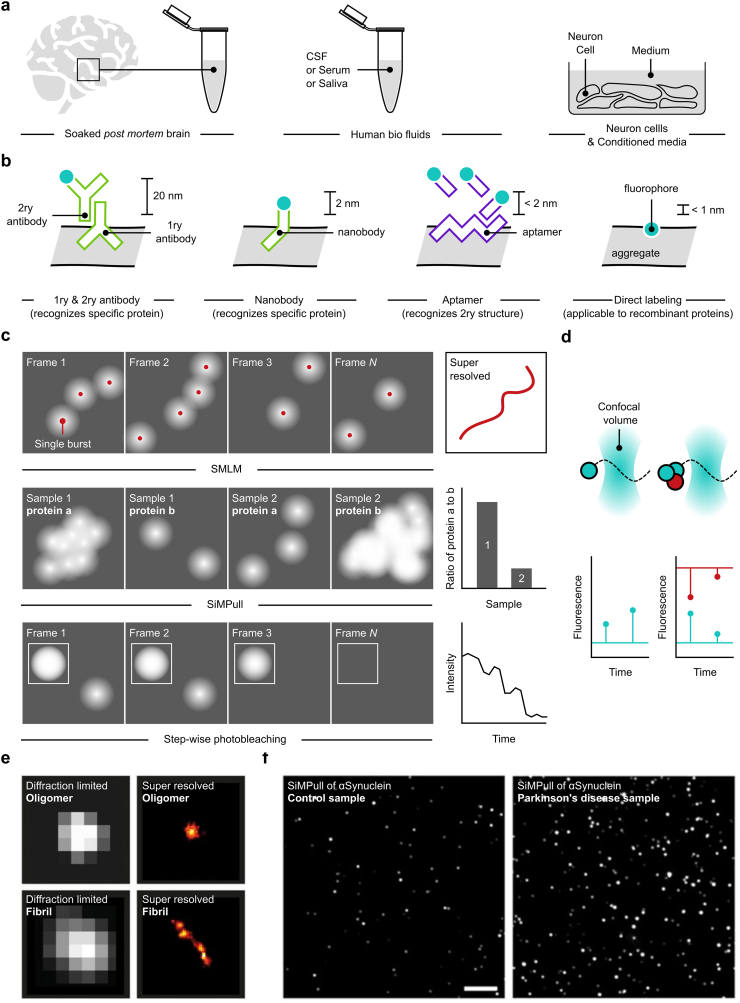
Principles underlying the operation of single molecule microscopy. (a) Schematic of the different human samples containing minute amounts of soluble aggregates which are compatible with single molecule microscopy. (b) Schematic of the different mechanisms used in labelling soluble aggregates for imaging and characterization using single molecule microscopy. (c) Principle underlying DNA-PAINT, SiMPull and single molecule photobleaching. (d) Schematic diagram of FCS (TCCD) for monomers (left panel) and oligomers (right panel). (e) oligomers and fibrils of amyloid aggregates super resolved using DNA-PAINT (reproduced with permission from (Whiten et al., 2018a)). (f) SiMPull of αS aggregates in postmortem brain punches from patients with and without PD (reproduced with permission from (Je et al., 2017)).

Single Molecule Localization Microscopy (SMLM) belongs to a larger family of microscopy techniques, known as super resolution microscopy, which can resolve biological structures smaller than the diffraction limited of light (below 200 nm) (Betzig et al., 2006; Hell and Wichmann, 1994; Klar and Hell, 1999; Hell, 2007; Rust et al., 2006). As opposed to conventional wide-field fluorescence microscopy which relies on exciting and imaging all fluorophores in a single frame, in SMLM, a small subset of fluorophores are excited and imaged at once resulting in spatially-distant, individually-resolvable single molecules whose centroids can be calculated with nanometer precision (Yildiz and Selvin, 2005). By recording the accurate positions of these centroids in another image and repeating the excitation, visualization and localization of a new set of sparse fluorescent molecules for several thousands of frames, a super-resolved image can be created for the underlying biological structure. The temporal separation (*i.e.* on/off switching) of fluorescent molecules between subsequent frames is key to super resolution imaging and there are several ways to achieve this. The first, known as Stochastic Optical Reconstruction Microscopy (STORM), uses enzymatic oxygen scavengers and high laser powers to deplete all fluorescent, photo switchable molecules in a field of view and re-activate a small, random subset each frame. Due to the use of oxygen scavengers and high laser powers, each fluorescent molecule does not necessarily photobleach immediately after being first excited but is rather promoted to a triplet state from it can be recycled and excited for several times. The second technique, known as Photo Activatable Localization Microscopy (PALM), differs from STORM in the use of medium to high laser powers in the absence of an oxygen scavenging system together with photoactivatable dyes. This configuration ensures all molecules emit only once before photobleaching (Bates et al., 2007; Huang et al., 2009; Heilemann et al., 2008; Heilemann et al., 2009). Both STORM and PALM have several limitations the most important being the small number of different protein targets that can be imaged in a field of view. Although several strategies were developed to overcome this problem, their implementation is complicated and would not allow more than 4 different protein targets to be imaged in a sample (Zhang et al., 2015). Provided several tens of proteins comprise the aggregation proteome, a more versatile imaging method is required. DNA Point Accumulation for Imaging in Nanoscale Topography (DNA-PAINT) was developed to overcome this limitation and is described in detail below (Jungmann et al., 2010; Jungmann et al., 2014; Schnitzbauer et al., 2017) (Fig. 1c).

### 2.2 Principle of DNA-PAINT

In DNA-PAINT, the target of interest is stained with a primary antibody, nanobody or aptamer (a small DNA quadruplex which recognizes a specific region on the protein of interest) conjugated with short, single-stranded DNA oligonucleotides known as ‘docking strands’. A solution of complementary fluorophore-conjugated ‘imager strands’ is flowed on top of the sample, some of which intermittently bind to their complementary docking strands. When the sample is imaged under Total Internal Reflection (TIR) of the excitation beam, only the bound fraction of all imager strands is detected within a shallow, a few hundred nanometers thick region above the coverslip. Using TIR illumination, the bound fraction is detected as bright bursts on a dark background which can be detected and processed as previously described with high fidelity. By staining different proteins with different antibodies, nanobodies or aptamers conjugated with different docking strands and using different imager strands complimentary in sequence to the docking strands labeling the proteins of interest, it is possible to super resolve the nanoscopic spatial organization of a large number of different proteins in a biological sample. The performance of DNA-PAINT has been extensively quantified using ground-truth DNA origami structures folded into user defined geometries and proteins with well-defined ultra-structures such Actin, Tubulin and the Nucleoporins (Thevathasan et al., 2019; Schlichthaerle et al., 2019).

### 2.3 Methodological advances to DNA-PAINT

DNA-PAINT is an improved variant of the traditional PAINT which used non-DNA based binding activatable fluorophores, such as pFTAA (Ries et al., 2013) and ThT^48^. Nevertheless, these dyes are not bright, their binding kinetics cannot be well controlled and can only recognize beta-sheet structures rather than individual proteins. DNA-PAINT was conceived to circumvent these limitations by combining the specificity of immunostaining with the ease of controlling the binding dynamic of short complementary DNA oligonucleotides (Jungmann et al., 2014). Still, DNA-PAINT comes with several drawbacks, the most important the speed of imaging and exchange of the imager strands. In the recent years, several clever strategies were developed to overcome these two problems. A straightforward solution to acquiring more data within a shorter time frame is to increase the size of the detection chip used for imaging. Widely available scientific Complementary Metal Oxide Semiconductor (sCMOS) cameras, boosting a 4 times larger detection area than the traditionally used electron multiplying Charge Coupled Detectors (emCCD), have been recently used in several studies to image a modestly larger number of single molecules in each frame (Stehr et al., 2019; Strauss and Jungmann, 2020; Strauss et al., 2018). Limiting the usability of this technology is the Gaussian profile of the excitation beam which although can be considered flat in the middle, decays rapidly towards the peripheries resulting in patches across the field of view where the localization precision is significantly deteriorated. To address this problem, a refractive beam shaping optic was recently reported to convert a Gaussian excitation beam into a homogenous flat-top beam that is compatible with TIR-based DNA-PAINT imaging and that boosts imaging speed and quantification across the entire chip (Stehr et al., 2019).

The major hurdle to overcome with regards to speed is the inverse relationship between the concentration of imager strands (corresponding to background intensity) and precision of localizing single molecules. This caveat limits the useable concentration of imager strands to approximately 1 nM which corresponds to a blink density of 0.005 blinks/μm^2^ per frame. Given the theoretical limit to the blink density is approximately 3 blinks/μm^2^ per frame, substantial improvements to the imaging speed can be accrued by developing strategies to increase the blink density. The first strategy addressing this gap is the use of docking strands that are labelled with donor fluorophores capable of transferring energy to the acceptor fluorophores conjugating imager strands – a configuration known as FRET-PAINT. When the donors are excited, emission from the acceptors are only detected whenever the imager strands are bound to their complementary docking strands within the TIR field (Auer et al., 2017). Limiting the emission to a few nanometers above the coverslip allows the use of high concentrations of imager strands reaching 1 μM corresponding to a high blink density approaching the theoretical limit. Nevertheless, the inefficient transfer of energy results in a 20% lower photon count compared to direct excitation and emission which results in a higher localization uncertainty, therefore, partially defying the purpose it is utilized for. A second more promising strategy is to shorten the time interval between one binding event and the other by using concatenated docking strands to allow more imager strands bind during the same duration. Expectedly, the improvement in speed is directly proportional to the number of concatenated docking strands. A modest 5 folds' improvement to the imaging speed corresponds to a 30 to 40 base pairs' (approximately 10 to 14 nm) longer docking strand which significantly deteriorates the localization accuracy. A clever twist which partially overcomes this problem is the development of overlapping docking strands; oligonucleotides which cannot only be stacked back-to-back but ones that are length-wise symmetric and, therefore, their concatenation results in a disproportionate larger number of binding sites (Strauss and Jungmann, 2020). A 5 folds' improvement to the imaging speed was demonstrated using 19 base pairs' (approximately 6.5 nm) long docking strands which although is a significant upgrade to concatenated strands it still does not allow super resolution imaging with molecular resolution. There is an opportunity to increase the number of binding events per unit duration whilst maintaining the spatial resolution below 5 nm using the so-called ‘super beacons’; fluorophores which are quenched in solution but emitting when bound to the target of interest (Pereira et al., 2020). Owing to the complexity in designing these foldable imager strands, developments in super-beacons are still undergoing. The last final approach used to increasing imaging speed is by decreasing the dwell time of the unbound imager strands through modulating their hybridization kinetics. A simple way to achieve this is to optimize the sequences of both the imager and docking strands or, to more broadly, use buffers with a high salt concentration (*e.g.* 75 mM MgCl_2_). Combining these two solutions can result in a 10 folds' increase in imaging speed corresponding to an 8 h' imaging time of a 1 mm × 1 mm field of view (Schueder et al., 2019).

With regards to increasing the speed of exchanging different imager strands to image multiple protein targets, limited improvements have been developed beyond manual exchange. Nevertheless, in an effort to push cost-efficient, fully-automated exchange DNA-PAINT, a versatile LEGO-based syringe pump system that is easily-interfaceable with widely available microscopy control software programs (*e.g.* micromanager) was developed and applied to DNA-PAINT imaging of 5 different ground-truth protein targets (Almada et al., 2019).

Additional technological advances were made to DNA-PAINT that further allowed it to extract structural information that were not conventionally available. Of these advances the development of spectral PAINT (sPAINT) (Lee et al., 2018); a technique which broadly allows different fluorophores, labeling different structures of interest, to be simultaneously identified and, therefore, push the speed of conventional PAINT imaging by several folds. sPAINT relies on placing a diffraction grating before the detector to split the incoming image in two halves: one half containing the single molecule bursts from which location information can be extracted with nanometer precision and, the second, containing the spectrum of each molecule which is exclusive to the emitting fluorophore. This versatile method does not only allow different molecular machines to be simultaneously localized with nanometer precision, but, also, for the action of achromatic fluorophores against different chemical or environmental changes to be traced at the nanoscale.

Amongst the most desired features in super resolution imaging is the ability to perform molecular counting at the nanoscale. In STORM and PALM this capability is, by far, immature due to the sporadic nature of the activation of each fluorophore. Quantitative PAINT (qPAINT) leverages the knowledge of the DNA binding kinetics to count the number of subunits comprising a nanoscopic assembly. Research in this domain is active and the mathematical operations developed for counting using PAINT vary in complexity and accuracy from simple multiplication of the number of binding events with a proportionality constant to retrieve the number of targets to more complex mathematical auto correlation of the temporal signal resulting from the binding events (Fricke et al., 2015; Karathanasis et al., 2017; Stein et al., 2019).

### 2.4 Application of DNA-PAINT to imaging protein aggregates

The multi-faceted developments made to DNA-PAINT in the recent years rendered the technology capable of tracing nanoscopic structural and organizational changes to the aggregates of misfolded protein in health and disease. The exquisite single molecule sensitivity of DNA-PAINT allows aggregates at sub picomolar concentrations to be easily detected. This allows a variety of different samples to be potentially imaged, including: human and animal secretions (*e.g.* Urine, Saliva), Cerebrospinal Fluid (CSF), blood serum, buffer soaked *post mortem* human brains, induced Pluripotent Stem Cells (iPSCs) (Whiten et al., 2018a; De et al., 2019) (Fig. 1a). Each of these samples is complicated to image for one reason or another and therefore careful optimization of sample preparation is a fundamental prerequisite to the high fidelity imaging of aggregates using DNA-PAINT. As an example, biofluids, such as the CSF, blood serum and buffer soaked *post mortem* human brains would often need to be mounted on chemically treated coverslips prior to imaging. Chemical treatments vary from one sample to the other and is often dependent on the balance between the proportion of aggregates to be adsorbed on the surface and the amount of non-specific binding of the imager strands to the glass surface that can be tolerated and which can contribute to a false positive signal during post processing. Treatments with surfactants (*e.g.* Tween) or acids (*e.g.* Aspartic acid) are common in the literature (Schnitzbauer et al., 2017). Although the effect of different chemical treatments on the non-specific binding of the imager strands is relatively well studied, however, to date, the effect of these treatments on the adsorption of aggregates of different structural isoforms remains unknown.

Similarly, the choice of the staining molecule requires a compromise between specificity and localization accuracy (Fig. 1b). Aptamers are the smallest non-antibody staining regents available and were recently used for DNA-PAINT imaging of aggregates in the CSF of diseased patients (Whiten et al., 2018a). These molecules are single stranded oligonucleotides folded into hairpin loops and are intrinsically limited in the confirmations they can adopt. Despite their small size and the positive effect of this on the localization accuracy, the largest drawback of these molecules, however, is their non-specificity which lends their use in staining aggregates inside samples with a large proteome (*e.g.* cells and blood serum) not optimal. Affimers are the smallest protein affinity reagents (approximately 2 nm wide) that are isolated from phage display libraries (Schlichthaerle et al., 2018). The small size, ease of isolation and high specificity of these molecules render them ideal candidates for use in super resolution microscopy, and particularly, DNA-PAINT. Although these molecules were used in imaging Actin, no literature suggests their use for imaging aggregates, and especially, those of different sizes (*i.e.* monomers, oligomers and fibrils), different structural isoforms and different stoichiometries. Nevertheless, these molecules were recently reported as alternatives to antibodies for the detection of amyloids in blood serum which suggests their potential use as suitable staining reagents for DNA-PAINT. Nanobodies, or single domain antibodies, are similar in size to affimers and have been extensively used in SMLM (Szymborska et al., 2013; Ries et al., 2012). The complexity in using nanobodies is that their isolation first requires the immunization of animals which is a long and expensive process (Harmsen and De Haard, 2007). More recently nanobodies to bind protein aggregates have been designed using a computational approach followed by testing of the highest affinity structures. This has resulted in high affinity nanobodies to amyloid beta (aβ) (Aprile et al., 2017; Aprile et al., 2020; Limbocker et al., 2020), one of which has been demonstrated to allow super-resolution imaging of aggregates. The suitability of nanobodies for DNA-PAINT imaging of protein aggregates has not been studied to date. Antibodies are the most versatile of these staining systems given their broad historical and commercial uses but come with the major drawback of introducing a large linkage error (approximately 20 nm) which significantly deteriorates the localization accuracy. Commercially available antibodies can detect monomeric, oligomeric and fibrillar forms of amyloid beta, alpha synuclein (αS) and tau misfolds in a variety of biological systems. The broad availability of antibodies is an important advantage, however, the linkage error these introduce is a major concern, particularly, when the species of interest are the small, toxic oligomers. An alternative to antibody-based immunostaining that is only applicable to cellular systems is direct labeling using enzymatic mediators, such as HALO- or SNAP- tags (Keppler et al., 2003; Los et al., 2008). DNA-PAINT imaging deploying these tags was performed on biological machineries with well-known ultra-structures (Schnitzbauer et al., 2017), however, DNA-PAINT imaging of protein aggregates was not demonstrated, to date, due to the complexity in characterizing the performance of these systems on the large variety of possible structural isoforms of protein aggregates.

### 2.5 Challenges in the application of DNA-PAINT to imaging protein aggregates in thick biological specimens

The application of DNA-PAINT to imaging aggregates in biofluids is more straightforward compared to cells, tissues, organoids and assembloids. Multiple factors, ranging from the technical complexity in exciting thin sections across the sample to the limited ability to detect single molecules across scattering media and correcting for optical aberrations, render this technology incompatible with imaging samples thicker than 1 μm. Despite this hurdle, imaging aggregates inside these samples is of supreme importance to dementia research, particularly in better visualizing the aggregation pathway and understanding its complex role in disease progression. There are several distinct solutions to this limitation. The first using STORM; a SMLM technique introduced earlier and which has the distinct advantage of allowing deeper imaging albeit at the expense of increased localization precision, translating to lower image resolution, and the inability to perform high quality super resolution imaging for more than 2 protein targets at a time. Despite these limitations, STORM is compatible with 3D imaging in thick samples, up to 5 μm in depth, through a number of methods which engineer the appearance of the single molecule bursts (technically known as the point spread function) to encode the axial, in addition, to the lateral position of each molecule (Shechtman et al., 2015; Huang et al., 2008; Y L, 2018). A second solution is to use lattice light sheet microscopy; an improved variant to the traditional light sheet microscopy which is capable of producing thin (100 nm) sections across thick samples (Chen et al., 2014). Although lattice light sheet was demonstrated on a wide range of cellular samples, its conception is expensive and complicated limiting its use to a few research labs worldwide. A third less complicated and more affordable solution combines spinning confocal microscopy with DNA-PAINT (Schueder et al., 2017). Confocal microscopy is a key imaging technology in biology owing to its ability to produce appropriately thin (<1 μm) optical sections across thick biological samples. Despite its simplicity and enormous power, it remains a diffracted-limited method. Combining DNA-PAINT with confocal microscopy leverages the strength of DNA-PAINT in resolving biological structures beyond the diffraction limit and confocal microscopy in allowing facile optical sectioning through the thick specimens of interest to dementia research. The fourth and final solution is to use, as previously discussed, non-fluorogenic imager strands (*i.e.* those which only fluoresce upon binding to their complementary docking strands and, therefore, target of interest) to avoid excessive background signal in the axial dimension. Super beacons and FRET pairs are two initially tested non-fluorogenic imager strands which were demonstrated with ground-truth structures (Auer et al., 2017; Pereira et al., 2020). It is worth re-iterating that the application of advanced imaging technologies to dementia research is, yet, at its infancy and although the previously mentioned solutions have been thoroughly tested with different samples and biological structures, they have not been applied, to the best of our knowledge, to imaging aggregates in complex specimen such as tissues and organoids.

### 2.6 Alternative single molecule microscopy modalities

The enormous power of SMLM, and particularly its high resolution variant DNA-PAINT, comes, in some cases, at the expense of complicated sample preparation protocols and technologically advanced optical setups which can form a barrier to its broad utility as well as customization for imaging the wide variety of aggregate isoforms' and biological specimens available. Furthermore, SMLM is suited for visualizing the nanoscopic spatial organization and steady-state structural heterogeneity of protein aggregates, but due to limitations in sample preparation and imaging speed, studying the dynamic interaction of the monomers, oligomers and fibrils with one another and the rest of the proteome within the cellular milieu is not possible. Fluorescence Correlation Spectroscopy (FCS) is an alternative technique which, although cannot image single molecules, has single molecule sensitivity and can detect inter- and intra-molecular interactions (Magde et al., 1972) (Fig. 1d**)**. In FCS, a laser is directed through an objective lens and into a specimen to form a tight spot, commonly referred to as the confocal volume, through which molecules can diffuse in and out. When the individual molecules of interest are labelled with a single fluorophore and allowed to diffuse within the confocal volume, a signal is detected. Monitoring the signal over time and mathematically processing it can quantify the size of the these molecules, how fast they diffuse and their concentration. Additionally, if each, or different, molecule(s) are labelled with two different fluorophores, the inter- and intra- molecular interactions can be quantified. FCS has extensively been utilized to quantity molecular interactions *in vitro* and *in vivo* (Schwille et al., 1999; Banks and Fradin, 2005; Eggeling et al., 2009; Petrášek and Schwille, 2008) as well as used for studying the growth kinetics of amyloid aggregates (see Section 3). The distinct advantage of FCS over SMLM is its ability to quantify molecular dynamics in real-time and in solution or inside the tubular and lamellar organelles of the cell (J R et al., 2009; Unsay et al., 2018) and using a wide array of labelling and immunostaining techniques. Combined with its cost-effectiveness and ease of assembly (Ambrose et al., 2020), confocal FCS is a viable solution for answering some questions related to the kinetics of aggregation.

The last single molecule microscopy technique to be discussed as part of this review and which is highly relevant to imaging and characterizing protein aggregates is the single molecule pull down assay (SiMPull) (Jain et al., 2012) (Fig. 1c). The proteins amyloid beta, αS and tau interact in complex ways and form heterogenous aggregates of different compositions and stoichiometries. The role of aggregate composition in the progression of dementia, synucleinopathies and tauopathies is unknown. Contributing to this knowledge gap is the lack of characterization tools, with high, single molecule, sensitivity and which can easily report the relative composition of the different proteins within the individual aggregates. SiMPull is a versatile method that can be used to measure the relative composition of different proteins in a sample of aggregates. In SiMPull, protein aggregates are captured using a ‘capture antibody’ on a glass surface. The capture antibody recognizes a specific protein (*e.g.* αS) of each aggregate. Aggregates not containing the specific protein to be captured, and therefore unbound, are washed off the surface. A set of detection (primary and secondary) antibodies are then used to report the presence and abundance of the protein of interest. The prepared sample is then imaged in widefield, similar to SMLM, and information on the relative amount of each protein for hundreds of different aggregates can be quantified from the emission intensity in a single frame. To measure the relative amount of different proteins in the same sample, different capture and detection antibodies. In addition, negative and positive controls need to be imaged under the same conditions to ensure the intensities observed for the sample of interest are accurate and not a result of an imaging artefact. SiMPull is a diffraction-limited imaging method which means that stoichiometry is reported on a number of aggregates and not on the level of single aggregates. Given that measuring the compositional markup of each soluble aggregate would be considered an important technological breakthrough in dementia research as well as a powerful tool to gain remarkable insight on how aggregates are different and how they drive disease, combining a super resolution microscopy technique, such as DNA-PAINT, with a technique that can measure the composition, such as SiMPull is an important pursuit. A correlative method allowing these measurements to be made simultaneously is qPAINT as previously described (Jungmann et al., 2016). Although qPAINT has been applied to quantifying the stoichiometry of several protein complexes, to the best our knowledge, it has not been demonstrated on protein aggregates.

Although SiMPull is quite powerful when coming to quantitate the relative abundance of proteins between different samples, it fails to produce an accurate, absolute readout of subunit counts in individual aggregates. An alternative method which overcomes this hurdle is single molecule step-wise photobleaching (Ulbrich and Isacoff, 2007; Das et al., 2007). In this method, low concentrations of the complex of interest are imaged for a few hundreds of frames until they photobleach. Examination of the intensities of these complexes reveals a characteristic ‘step-wise’ decrease in intensity over time. Since the photobleaching of a single molecule manifests itself as a single step in its intensity trace, counting the number of steps in the intensity trace of the photobleached complex corresponds to its absolute stoichiometry subject to corrections in the labeling efficiency. This method was extensively used in measuring the stoichiometries of many protein assemblies including amyloid aggregates (Zijlstra et al., 2012; Dresser et al., 2020).

## 3 Biological insights from the application of single molecule microscopy to protein aggregates

In the previous section, we discussed three single molecule microscopy modalities: SMLM, which is used for super-resolving biological structures at the nanoscale, FCS, which is used for measuring dynamic changes and interactions within, or between, protein(s) and SiMPull, which is used for measuring the composition of protein complexes in different samples. In this section, we review the recent discoveries made as part of imaging or characterizing protein aggregates using the described single molecule microscopy modalities (Table 1).

**Table 1:** List of key studies performed on protein aggregates using single molecule microscopy ordered in the chronological order of molecular events in disease progression.

Authors	Year	Details	Reference
Kinetic characterization of protein aggregates			
Orte el al.	2008	Characterizing aggregates of the amyloidogenic PI3 kinase. Toxic oligomers are found not to change size as time progresses but that their mode of action is dictated by internal structural reorganization.	Orte et al., 2008
Roberti et al.	2012	Characterizing the elongation rate of αS aggregates *in vivo*	Roberti et al., 2012
Cremades et al.	2012	Charactering aggregates of recombinant αS. Oligomers are found to exhibit 3 distinct size ranges which correspond to 2 distinct structural confirmations; one that is persistent and stable another that is not. Long fibrils are found to disaggregate into the stable oligomers. Findings allow a thorough kinetic model to be built.	Cremades et al., 2012
Pinotsi et al.	2014	Using dual-colour STORM to image the elongation of amyloid aggregates from both ends.	Pinotsi et al., 2014
Horrocks et al.	2015	Single molecule characterization experiments are sped-up by the use of fast flow microfluidics. This advance allow the characterization of aggregates under various conditions.	Horrocks et al., 2015
Tosatto et al.	2015	Characterizing differently mutated aggregates. Findings suggest structural properties, as opposed to size, regulate disease progression.	Tosatto et al., 2015
Pinotsi et al.	2016	Characterizing the seeding and toxicity of monomeric and oligomeric αS species *in vivo*	Pinotsi et al., 2016
Steady state characterization of protein aggregates			
Schierle et al.	2011	Observing the varied structural morphologies of aβ aggregates *in vitro* and *in vivo*	Kaminski Schierle et al., 2011
Zijlstra et al.	2012	Counting of the number of subunits constituting αS oligomers using step wise photobleaching	Zijlstra et al., 2012
Horrocks et al.	2016	The amyloid sensitive ThT fluorophore is used in detecting recombinant and human oligomers and fibrils in various specimen.	Horrocks et al., 2016
Fritschi et al.	2017	Comparison of the seeding propensity in the brain and CSF of Alzheimer's disease patients using STORM.	Fritschi et al., 2017
Lee et al.	2018	Surface hydrophobicity of individual aggregates is quantified on the nanoscale using the Nile Red fluorophore.	Lee et al., 2018
Whiten et al.	2018	Nanoscopic aggregates are resolved using aptamer based DNA-PAINT in iPSCs harbouring the SNCA triplication.	Whiten et al., 2018a
De et al.	2019	Nanoscopic aggregates in the CSF of diseased and control patients are super resolved. No difference in the concentration of aggregates detection for diseased patients compared co healthy controls. However, the median length of the aggregates was found larger in patients with Mild Cognitive Impairment compared to disease and control patients.	De et al., 2019
Dresser et al.	2020	Counting of the number of subunits constituting aβ oligomers using step wise photobleaching	Dresser et al., 2020
Characterizing the interaction of amyloid aggregates with the neuronal proteome			
Whiten et al.	2018	The interaction of the chaperones (CLU and α2M) with aggregates is characterized and found to exhibit different binding properties to oligomers of different sizes.	Whiten et al., 2018b
Ludtmann et al.	2018	Interaction of αS oligomers with ATP synthase.	Ludtmann et al., 2018
Ye et al.	2020	Effect of Tau and aβ ubiquitination on the oligomerization process is studied.	Ye et al., 2020

### 3.1 Nanoscopic characterization of protein aggregates

The earliest single molecule experiments performed on aggregates used the fluorophore ThT which only binds to cross β sheet aggregates and whose quantum yield (*i.e.* fluorescence emission) increases up to 4000 folds upon binding (Horrocks et al., 2016). In these experiments, the use of ThT was combined high sensitivity emCCD cameras to establish three important findings: the first that ThT can recognize the toxic and inflammatory oligomers and fibrils and not the disease unrelated monomeric species, the second that concentrations as low as 10 pM of these aggregates can be readily detected and, the third, that this method can be applied to human biofluids. To demonstrate the applicability of this method, aggregates in the CSF of 18 patients with Parkinson's Disease (PD) and 18 healthy controls were imaged and counted. The median number of soluble aggregates in the PD patients was found to be roughly twice as large as that in healthy controls. Furthermore, when the frequency distributions between the sets of samples was compared, the difference was found to be statistically significant (*p* < 0.001), compared to concentration distributions for the same samples obtained using an αS enzyme-linked immunosorbent assay (ELISA). Although these findings establish the prominence of diffraction-limited ThT imaging as a powerful, versatile and quick tool for quantifying the number of soluble aggregates secreted in biofluids under health and disease and, therefore, as an ultra-sensitive method for the early diagnosis of dementia, large variations in the number of aggregates between different patients render this technique practically impossible to use as an explorative tool for studying aggregation in individual samples with confidence. These experiments disclosed the need for a more powerful technology that can better resolve the nanoscopic aggregates and allow the extraction of additional geometrical parameters that can be correlated with disease progression in individual patients. In principle, ThT can be used to super resolve the structure of individual amyloid aggregates, however as it emits in the ultra violet, a wavelengths regime in which the quantum efficiency of available detectors does not exceed 70%, and with a low quantum yield which affects the localization precision, it is not possible to use it to obtain high resolution images of the ultrastructure of these aggregates. Other fluorophores such as the luminescent conjugated oligothiophene p-FTAA (Ries et al., 2013) or the improved version of ThT, ThX, were recently used (Needham et al., 2020), however, with regards to p-FTAA, no studies were performed demonstrating its applicability to aggregates from human samples and for ThX, a 100 nm resolution was shown which is not enough given the length of soluble aggregates *in vivo* could be smaller.

The earliest experiments circumventing the combined problems encountered with ThT and p-FTAA used STORM (Kaminski Schierle et al., 2011). In these experiments, the aβ peptides (1–40) and (1–42) were directly labelled with Fluor488 and imaged *in vitro* as well as *in vivo* which revealed the striking differences in the ultra-structures exhibited by aβ oligomers and fibrils under different environmental conditions. These measurements were later extended to αS aggregates to show, with nanoscopic resolution, that they elongate through nucleation from both ends with largely varying nucleation rates (Pinotsi et al., 2014). The same method was finally applied to the CSF and postmortem brain of patients diagnosed with Alzheimer's disease to show that the aβ peptides (1–40) has a high propensity to aggregate in the brain, but not the CSF, sample (Fritschi et al., 2014). Despite these breakthroughs, STORM, as previously discussed, is limited in its multiplexing capabilities rendering multicolour experiments not possible. To ameliorate this limitation, AD-PAINT was developed and first used to image the structure of protein aggregates in iPSCs from PD patients harbouring a triplicate of the αS gene (SNCA) and healthy controls (Whiten et al., 2018a). In this study, the number of aggregates per field of view as well as the number of localizations and length of each aggregate were measured. All of the 3 measurements were on average higher in the iPSCs harbouring the SNCA triplication compared to the healthy controls. Strikingly, however, the number of aggregates showed the most statistically significant difference (*p* < 0.0001) compared to the number of localizations (*p* < 0.001) and length (*p* < 0.05). Although these findings are surprising, as it was earlier expected that the length measurements would better correlate with disease pathology, nevertheless, these experiments disclosed the need to investigate other structural attributes (*e.g.* protein composition of each aggregate) which are better correlated with the presence of disease.

The composition of soluble protein aggregates and its role in neurodegeneration is a poorly studied aspect due to the lack of suitable characterization techniques. Still the composition might be an important structural and functional regulator of the aggregation pathway. We have previously discussed SiMPull as a simple, yet powerful, tool for measuring the composition of protein complexes with single molecule precision. In a recent study, αS SiMPull was applied to *post mortem* brains of humans diagnosed with PD as well as healthy controls to image endogenous αS aggregates (Je et al., 2017). To make these measurements, aggregates were sandwiched between 2 antibody systems: a primary and secondary capture antibodies which hook the aggregates to a Polyethylene glycol (PEG) coated coverslip and, another, primary and secondary detection antibodies of which the latter is conjugated to a synthetic fluorophore as a reporter. To establish the accuracy of SiMPull in detecting minute amounts of endogenous αS and ensure nonspecific binding of the antibody systems is minimized, four experiments were performed. In the first experiment. The presence and absence of recombinant αS, prepared from monomeric species after 5 days of incubation, was measured. At concentrations as low as 75 pg/μL the SiMPull assay showed clear differences when compared to the negative control (*i.e.* sample with no aggregates). Various studies have shown recombinant αS to be structurally and functionally different from endogenous αS. To ensure the SiMPull assay can detect the endogenous, disease-implicated aggregates, two sets of 293 T cells were prepared: the first with αS over expressed and the second in which the SNCA gene knocked out using the CRISPR/Cas9 gene editing technology to thus yield one set of cells with αS aggregates and another without. In this second experiment, the cells where lysed and the lysates at a low concentration of 10 ng/μL were collected and imaged. Lysates containing the aggregates showed a much higher signal compared to those not containing the aggregates. In the third experiment, the minimal concentration of lysates from cells with endogenous aggregates was established. Four lysate concentrations of 10, 25, 50 and 100 ng/μL were tested and aggregates were still detectable at 10 ng/μL compared to the lysates from the knockout cells not containing αS aggregates. Finally, in the fourth experiment, monomeric, oligomeric and fibrillar aggregates were prepared recombinantly and imaged using the SiMPull assay by truncating the full length secondary detection antibody and using a relatively low degree of labelling to prevent steric hinderance between the antibodies. High intensity and elongated spots were exclusively observed in the samples containing the oligomeric species which confirms the ability of the antibody system to recognize monomeric, oligomeric and fibrillar species of αS. Following these confirmatory experiments, *post mortem* brain samples from the *substantia nigra* were obtained from 3 healthy controls and 3 individuals with PD to characterize the αS aggregates present within. This experiment confirmed 2 important results: the first that the number of αS aggregates in the PD samples is approximately 3 folds higher than in the healthy controls (distributions are statistically significant at *p* < 0.0001) and, the second, that the fraction of oligomeric species is also higher (statistically significant at *p* < 0.05). This important study establishes the use of SiMPull as a robust method to measure picomolar protein concentrations forming the soluble aggregates.

Augmenting the SiMPull measurements is the use of step-wise single molecule photobleaching to quantify the absolute molecular stoichiometry of αS aggregates. In one study, the recombinant wild type and mutant (A140C) forms of αS were expressed, purified, directly labelled and incubated to produce singly labelled oligomeric species and observed at low concentrations until photobleached. Analysis of the photobleached traces strikingly revealed a fixed molecular composition of 31 subunits, which is in good agreement with measurements performed using FCS^86^. In another study, the same method was applied to measure the stoichiometry of the aβ555 peptide *in vitro* and found that oligomers exhibit, on average, a much smaller number of subunits (~2) although the stoichiometric distribution is broader than that of αS aggregates (Dresser et al., 2020).

Complementing these studies is the use of sPAINT to map the surface hydrophobicity of single αS aggregates at the nanoscale (Lee et al., 2018). Surface hydrophobicity is an important structural characteristic which regulates the interaction of aggregates with biological membranes and, eventually, their ability to permeabilize them (*i.e.* their degree of toxicity). To measure the surface hydrophobicity of each aggregate, the solvatochromatic dye Nile Red, which changes its fluorescence spectrum upon transient binding to aggregates with different surface hydrophobicities, was used. Recombinant αS aggregates were prepared from 70 μM of monomers incubated at 37 degC and extracted at different time points (1, 3, 9, 24 and 48 h). In the earlier time points, aggregates, mostly globular, showed increased hydrophobicity compared to aggregates from the later time points which were mostly fibrillar. These findings confirmed previous reports that the smaller species are more toxic (*i.e.* more membrane permeable) being more hydrophobic. Surprisingly, however, when the long, fibrillar aggregates were incubated at 1 μM for over a month, extracted and imaged, they showed increased hydrophobicity, and toxicity, over time suggesting a need for inhibiting the growth of these species to prevent their implication in disease progression. Furthermore, these aggregates exhibited 2 populations only: monomeric and fibrillar species with the medium-sized oligomeric species being absent from the analyzed samples. This suggests that the intermediate oligomers are only formed at high seeding concentrations only. Finally, by plotting the hydrophobicity heterogeneity, defined as the mean hydrophobicity divided by the variability in hydrophobicity, 3 distinct populations were unveiled which, when compared to ThT imaging, were found to correspond to the monomeric, oligomeric and fibrillar species. Further experiments were also performed to identify the chemical changes underlying variations in hydrophobicity, such as digesting the aggregates using Proteinase K (an enzyme which removes hydrophobic amino acids).

A final important study focused on examining the nanoscopic differences in the soluble aggregates secreted in the CSF of healthy and diseased patients diagnosed with either late stage AD or mild cognitive impairment (MCI) (De et al., 2019). By imaging the aggregates using AD-PAINT, the authors found that the number of aggregates per field of view is similar between healthy controls, MCI and AD patients, whilst the median length of aggregates was larger for the MCI patients compared to both the AD patients and healthy controls which, together with findings from other ensemble assays, suggests that aggregates change in size and toxicity mechanism during disease progression. In particular, by also using atomic force microscopy to image the aggregates, the study showed that protofibrillar aggregates formed during the development of AD caused inflammation, which in turn can cause memory loss (Hughes et al., 2020).

### 3.2 Measuring the aggregation kinetics at the single molecule level

We have, thus far, described studies in which the number of localizations, length, composition and hydrophobicity of individual aggregates as well as the number of different proteins composing these in very small amounts can be accurately quantified with single molecule precision and across a wide range of samples, from CSF to *post mortem* brain punches. Nevertheless, all these studies focus on the steady-state structural descriptors of these aggregates which are particularly important in characterizing, diagnosing and identifying the origins of dementia. Single molecule imaging is not suited, however, for understanding how aggregates evolve over time and under various conditions as well as measuring the effects of this on disease progression. FCS emerges as an alternative tool that can measure aggregation kinetics with single molecule precision and in real-time.

The earliest experiments were performed almost a decade ago using 2 colour FCS, or as known at that time by Two Colour Coincidence Detection (TCCD), to study the aggregation of the amyloidogenic PI3 kinase (Orte et al., 2008). To perform these studies, the SH3 domain of PI3 was mutated in the N-terminus (M1C) to permit direct maleimide labelling with equimolar concentrations of either Alexa 488 or Alexa 647. The labelled species were mixed and aliquots extracted at different time points for analysis using TCCD. Findings revealed the most toxic species to be differently-sized nanoscopic oligomers. Surprisingly, although the size of the oligomers does not change over time, they become more stable which suggests that aggregation proceeds by internal reorganization to form the cross beta sheets. Later the same method was used to characterize recombinant αS aggregates (Cremades et al., 2012). As opposed to the earlier experiments in which both fluorophores, Alexa 488 and Alexa 647, were simultaneously excited, only the Alexa 488 fluorophores were excited in this experiment and emissions from both fluorophores were collected to exploit, in addition to the size measurements, intra structural changes resulting from the transfer of energy between both fluorophores (*i.e.* single molecule FRET, smFRET). The experiments unveiled 4 distinct populations: the first, small aggregates (2 to 5 monomers) with medium FRET (0.4 to 0.7), the second, medium aggregates (5 to 15 monomers) with medium FRET, the third, medium aggregates with high FRET (0.6 to 0.9) and the fourth, large aggregates (15 to 150 monomers) with high FRET. The time dependence of each category was fitted to a single exponential and a growth rate was extracted accordingly. The growth rates for the oligomers belonging to the first and second categories were equal and so were these of the third and fourth categories suggesting structurally different aggregates. To further understand the discrepancy in the FRET efficiencies and growth rates, the obtained oligomers were subjected to Proteinase K digestion. Interestingly, the oligomer population exhibiting low FRET were less resistant to proteolytic degradation compared to the population exhibiting high FRET which suggests the former to lack a stable, persistent structure compared to the latter. Moreover, when formed fibrils are disaggregated they decompose into oligomers characterized by high FRET efficiencies. These findings point towards a general aggregation model starting by nucleation of the monomers into structurally-unstable oligomers which could reversibly elongate into structurally stable or unstable oligomers followed by fibrils that can only disintegrate into stable oligomers.

Due to the long data acquisition periods in the previous study (3 h per data point), limited investigations were made to understand the nature of the αS oligomers. To empower further investigations on the nature of these aggregates, TCCD was combined with a fast flow microfluidic system which was demonstrated to lower down the acquisition period to 5 min per data point (Horrocks et al., 2015). With this technological improvement, the effect of pH and ionic strength on the conformation of monomeric αS and the electrostatic stability of its oligomeric species was examined. These experiments revealed that the larger, more stable, low FRET oligomers are more susceptible to changes in ionic strength compared to the smaller, less stable, high FRET oligomers which can be explained by shielding the additional charges and increasing the stability of the hydrophobic interactions. In a later study using the same technology, 3 mutated forms of αS (A53T, A30P and E46K) were studied for their ability to aggregate compared to the wild type originally examined (Tosatto et al., 2015). Three important findings were established in these experiments: first, that the kinetics of oligomer formation and stability correlated with the tendency of each mutant to form persistent β sheets, second, that the two mutants, A53T and A30P, showed increased standard deviation in their FRET measurements which indicates the presence of a heterogenous population of aggregates and, third, that the concentrations of the mutants and wild type were similar across all time points which suggests that the structural properties of these oligomers regulate disease progression as opposed to their absolute amount as previously thought.

STORM was also used in studying the aggregation dynamics of recombinant αS, both *in vitro* and *in vivo*. In one interesting study, the exogenous monomeric and fibrillar forms of αS were added to cultured neuron cells under two separate experiments. Fibrillar αS was found to trigger the nucleation of endogenous αS but not causing cellular death. However, monomeric αS was found to aggregate inside the cells causing death through the apoptotic pathway. Surprisingly though, addition of fibrillar αS in the latter case prevented cellular death pointing towards its protective role in neurodegeneration and in line with previous studies showing the oligomeric, and not the fibrillar, species to be neurotoxic (Pinotsi et al., 2016). In another similar study, recombinant αS were microinjected in HeLa cells and imaged below the diffraction limit using PALM. Aggregates were monitored over time and their elongation was estimated at 2 nm per minute which displays the power and exquisite resolution of SMLM in deciphering the nanoscale kinetics of amyloid aggregation *in vivo* (Roberti et al., 2012).

### 3.3 Studying the interaction of protein aggregates with the organelles and proteome of the cell at the nanoscale

In all of the above-mentioned studies, protein aggregates were studied in isolation. Obviously, the complex interactions of the aggregates with the neuronal organelles' network and the synaptic protein machinery regulates the progression of neurodegenerative disease. The combination of TCCD, particularly smFRET, and sPAINT has allowed, very recently, examination of the interactions of αS aggregates with key proteins and organelles implicated in neurodegeneration. In a first study, the interaction between the extracellular chaperones, Clusterin (CLU) and α_2_-macroglobulin (α_2_M), which enhance the clearance of amyloid aggregates and protect neuronal cells from proteotoxicity from endoplasmic reticulum (ER) stress, were studied (Whiten et al., 2018b). Despite the important role of these chaperones in resisting Parkinson's disease, the nature of their interactions with aggregates was poorly studied at the time. By labeling αS with the blue-excited fluorophore, Alexa 488, and CLU with Alexa 647 it was found that CLU was strongly associated with small oligomers (*i.e.* containing less than 15 monomers) and mildly associated with larger oligomers which suggests that CLU is able to bind to oligomers of a broad size range, from dimers to 30-mers. The binding of α_2_M to αS aggregates was also examined and, interestingly, were found to exhibit a linear variability to the oligomer size – unlike the binding of CLU. The use of TCCD was combined with sPAINT to visualize the regions to which CLU and α_2_M bind to αS aggregates. These correlative measurements disclosed the binding of CLU and α_2_M to the exposed hydrophobic surfaces of the αS aggregates. All in all, and in line with previous findings, CLU and α_2_M exhibit preferential binding to αS aggregates which is most likely due to structural attributes.

The same methodology was exclusively applied to the microtubule binding, Tau (Ye et al., 2020). Tau, together with aβ and αS, form a potent triad that is highly implicated in Alzheimer's and Parkinson's disease. Despite its importance in disease pathology, it has not been extensively studied as aβ and αS with single molecule sensitivity. In this study, the effect of structural post modifications made by the Ubconjugating enzyme, UBE2W, in the aβ and αS monomers, on their propensity to aggregate were examined. Ubiquitin modification was found to produce structurally distinct oligomers compared to their unmodified counterparts which were degraded in different numbers by the proteasomal machinery.

Finally, the interaction of the monomeric and oligomeric forms of αS with ATP synthase were studied using DNA-PAINT and a set of complementary biophysical techniques (Ludtmann et al., 2018). In this study, the oligomeric forms of αS were found to localize at the mitochondria impairing the complex I-dependent respiration, leading to the opening of permeability transition pore and triggering cell death in several disease models. This is an important finding as it relates the molecular interactions of amyloid aggregates with the mitochondrial machinery and explains its implication in the disease cascade.

## 4 Open questions and future directions

In this review, we covered the fundamentals and application of single molecule microscopy in imaging and characterizing protein aggregates. All work, done to date, is concentrated on understanding how misfolded proteins aggregate, what influences their aggregation and how are aggregates different between healthy and diseased individuals. Although these studies might seem comprehensive, protein aggregation is one of the most complex and important processes in brain biology. Evidence to the complexity of this process is the many failed drug trials targeting this pathway. Key questions related to the ubiquitous and enigmatic role of aggregation in dementia remain unanswered, amongst these:

1. What causes protein aggregation in sporadic Alzheimer's disease?
2. What is the relationship between aggregation and disease progression: does aggregation cause disease, disease cause aggregation or a complex feedback loop regulates both?
3. How do aggregates interact with the extensive organelle and proteome network inside the neurons and specifically at the synaptic terminals? And how does this sophisticated interactome lead to the progression, acceleration and delay of disease?

For many obvious reasons, no answers were obtained in response to these questions for the last 25 years. Owing to its applicability to a broad range of samples from human biofluids to organoids as well as unparalleled sensitivity and precision, single molecule microscopy has the potential to revolutionize our understanding and diagnosis of these fatal diseases and allow us to address these questions.
